# FUNCTIONAL WELLNESS AND OLDER MEN WITH HIV

**DOI:** 10.1093/geroni/igz038.2024

**Published:** 2019-11-08

**Authors:** Tonya N Taylor, Jeremy Weedon, Tarik Silk

**Affiliations:** SUNY Downstate Medical Center, Brooklyn, New York, United States

## Abstract

Aging-related stressors, such as changing physical function, poorly managed multimorbidity, increasing pill burden and social losses can diminish for some older adults with HIV the capacity for self-care. These challenges, however, can be improved by maintaining physical, cognitive and social function, or functional wellness. Using cross-sectional data from a men’s health study with younger and older men with and without HIV, we conducted general linear models to identify individual and clinical predictors for physical, cognitive, social and role function, as measured by the Medical Outcomes Study HIV Health Survey. We found that older HIV+ men had lower burdens of functional deficits compared to older HIV- men and younger HIV+ men and that across all models, depression, followed by diabetes, housing, and employment were predictive of functional wellness. Functional wellness for older HIV+ men is a multidimensional construct that includes optimizing internal and external resources to maintain healthy living and wellness.

